# Blood Cytokine Analysis Suggests That SARS-CoV-2 Infection Results in a Sustained Tumour Promoting Environment in Cancer Patients

**DOI:** 10.3390/cancers13225718

**Published:** 2021-11-15

**Authors:** Fien H. R. De Winter, An Hotterbeekx, Manon T. Huizing, Angelina Konnova, Erik Fransen, Bart ’s Jongers, Ravi Kumar Jairam, Vincent Van averbeke, Pieter Moons, Ella Roelant, Debbie Le Blon, Wim Vanden Berghe, Annelies Janssens, Willem Lybaert, Lieselot Croes, Christof Vulsteke, Surbhi Malhotra-Kumar, Herman Goossens, Zwi Berneman, Marc Peeters, Peter A. van Dam, Samir Kumar-Singh

**Affiliations:** 1 Molecular Pathology Group, Laboratory of Cell Biology & Histology, Faculty of Medicine and Health Sciences, University of Antwerp, Universiteitsplein 1, 2610 Wilrijk, Belgium; fien.dewinter@uantwerpen.be (F.H.R.D.W.); an.hotterbeekx@uantwerpen.be (A.H.); Angelina.konnova@uantwerpen.be (A.K.); bart.sjongers@uantwerpen.be (B.J.); ravikumar.jairam@uantwerpen.be (R.K.J.); vincent.vanaverbeke@uantwerpen.be (V.V.a.); 2 Multidisciplinary Oncologic Centre Antwerp (MOCA), Antwerp University Hospital, Drie Eikenstraat 655, 2650 Edegem, Belgium; manon.huizing@uza.be (M.T.H.); annelies.janssens@uza.be (A.J.); Zwi.Berneman@uza.be (Z.B.); marc.peeters@uza.be (M.P.); Peter.VanDam@uza.be (P.A.v.D.); 3 Biobank Antwerp, Antwerp University Hospital, Drie Eikenstraat 655, 2650 Edegem, Belgium; pieter.moons@uza.be; 4 Laboratory of Medical Microbiology, Vaccine and Infectious Disease Institute, University of Antwerp, Universiteitsplein 1, 2610 Wilrijk, Belgium; surbhi.malhotra@uantwerpen.be (S.M.-K.); herman.goossens@uantwerpen.be (H.G.); 5 StatUa, Center for Statistics, University of Antwerp, 2000 Antwerp, Belgium; erik.fransen@uantwerpen.be (E.F.); Ella.roelant@uza.be (E.R.); 6 Clinical Trial Center (CTC), CRC Antwerp, Antwerp University Hospital, University of Antwerp, Drie Eikenstraat 655, 2650 Edegem, Belgium; christof.vulsteke@azmmsj.be; 7 Center for Oncological Research (CORE), Integrated Personalized and Precision Oncology Network (IPPON), University of Antwerp, Universiteitsplein 1, 2610 Wilrijk, Belgium; debbie.leblon@uantwerpen.be (D.L.B.); lieselot.croes@azmmsj.be (L.C.); 8 PPES Lab Protein Chemistry, Proteomics & Epigenetic Signaling, IPPON, Department Biomedical Sciences, University Antwerp, Universiteitsplein 1, 2610 Wilrijk, Belgium; wim.vandenberghe@uantwerpen.be; 9 Department of Medical Oncology, AZ Nikolaas, Moerlandstraat 1, 9100 Sint-Niklaas, Belgium; willem.lybaert@telenet.be; 10 Integrated Cancer Center Ghent, Department of Medical Oncology, AZ Maria Middelares, Buitenring Sint-Denijs 30, 9000 Ghent, Belgium

**Keywords:** COVID-19, immune response, Th1, Th2, Th17, pro-inflammatory, anti-inflammatory, solid cancers, haematological cancers

## Abstract

**Simple Summary:**

SARS-CoV-2 infection has been associated with broad dysregulation of the circulating immune system and inflammation that could persist for a long period. Chronic inflammation has been shown to play a significant role in cancer development and progression. Here we showed that several immune-related factors were altered in cancer patients who had previously been exposed to the virus. Several of these immune factors observed to be elevated even after 3 months of infection are also tumour-promoting factors. The results of this study suggest that we need to pay more attention to cancer patients who have recovered from COVID-19 for any increased rate of cancer progression.

**Abstract:**

Cytokines, chemokines, and (angiogenic) growth factors (CCGs) have been shown to play an intricate role in the progression of both solid and haematological malignancies. Recent studies have shown that SARS-CoV-2 infection leads to a worse outcome in cancer patients, especially in haematological malignancy patients. Here, we investigated how SARS-CoV-2 infection impacts the already altered CCG levels in solid or haematological malignancies, specifically, whether there is a protective effect or rather a potentially higher risk for major COVID-19 complications in cancer patients due to elevated CCGs linked to cancer progression. Serially analysing immune responses with 55 CCGs in cancer patients under active treatment with or without SARS-CoV-2 infection, we first showed that cancer patients without SARS-CoV-2 infection (*n* = 54) demonstrate elevated levels of 35 CCGs compared to the non-cancer, non-infected control group of health care workers (*n* = 42). Of the 35 CCGs, 19 were common to both the solid and haematological malignancy groups and comprised previously described cytokines such as IL-6, TNF-α, IL-1Ra, IL-17A, and VEGF, but also several less well described cytokines/chemokines such as Fractalkine, Tie-2, and T cell chemokine CTACK. Importantly, we show here that 7 CCGs are significantly altered in SARS-CoV-2 exposed cancer patients (*n* = 52). Of these, TNF-α, IFN-β, TSLP, and sVCAM-1, identified to be elevated in haematological cancers, are also known tumour-promoting factors. Longitudinal analysis conducted over 3 months showed persistence of several tumour-promoting CCGs in SARS-CoV-2 exposed cancer patients. These data demonstrate a need for increased vigilance for haematological malignancy patients as a part of long COVID follow-up.

## 1. Introduction

Inflammation is one of the hallmarks of cancer. While chronic inflammation results in a predisposition to the development of specific cancers, cancer-related inflammation generally affects all aspects of malignancies, including proliferation and survival of malignant cells, angiogenesis, and metastasis [1,2,3]. Immune cell infiltration is observed in almost all tumours, ranging from subtle infiltrations detectable by only cell-type specific antibodies to gross accumulations of lymphocytes, macrophages, or mast cells [1,2,3]. These cell infiltrates secrete a repertoire of inflammatory proteins such as cytokines, which serve as important orchestrators of cancer–inflammation interactions. For instance, cytokines like interleukin (IL)-2, IL-12, and certain interferons might inhibit the growth of tumours. However, paradoxically, many cytokines support chronic inflammation thereby promoting tumour growth and influencing multiple aspects of cancer metastasis. Examples of such tumour-promoting cytokines are IL-6, tumour necrosis factor α (TNF-α) and transforming growth factor β (TGF-β), where several clinical trials targeting these cancer-promoting cytokines are ongoing [3,4,5].

Several cytokines, chemokines, and growth factors (CCGs) are also currently being utilized in cancer patient care as prognostic classifiers [6,7,8,9,10]. A comprehensive CCG analysis is thus essential to untangle the individual interactions in the understanding of CCGs’ cumulative role in tumour growth and metastasis. While each cancer is associated with a distinct repertoire of cytokines, broad similarities might be present between solid and haematological malignancies, especially in the immunocompromised state of the metastatic phase [11,12]. For example, angiogenic factors are generally accepted to promote further growth of solid tumours but are also now increasingly found to be involved in haematological malignancies [13,14]. However, to our knowledge, a very limited number of studies have employed a comprehensive, quantitative analysis of CCGs in studies of cancer [7,9,15].

In the host defence system against infections, cytokines also play an important role in clearing viral or bacterial pathogens. Cancer patients are more susceptible to infections due to immunosuppression caused by cancer-mediated immune factors as well as systemic treatment. Thus, not surprisingly, cancer patients, especially those with advanced disease or haematological malignancies, were shown to be at higher risk of developing severe COVID-19 [16,17,18]. For instance, in the initial outbreak in Wuhan, a more severe disease was reported in hospitalized cancer patients than non-cancer patients, with higher rates of ICU admission, requirement for invasive ventilation, and higher mortality [16]. These data are in agreement with another study that, after correction for age and other co-morbidities, reported more severe COVID-19 in cancer patients, particularly in haematological and lung malignancies as well as in all metastatic stage IV cancers [17]. However, not all studies have confirmed these data [19] and it remains possible that the increased mortality in cancer patients is inherently biased by comorbidities such as old age, obesity and diabetes, or inadequate therapy and follow up during the COVID-19 pandemic [20]. An equally relevant question is how CCGs are modulated in cancer patients after SARS-CoV-2 infection/exposure. Several pro-inflammatory cytokines observed to be massively upregulated in SARS-CoV-2 infection, such as IL-6 and TNF-α [21,22,23,24,25], are also tumour-promoting factors and therefore could alter the rate of cancer progression. It is also possible that already elevated levels of cytokines such as IL-6 and TNF-α in cancer patients offer protection in the initial stages of SARS-CoV-2 infection, offering a certain degree of immune-preparedness. To the best of our knowledge, only a few studies have investigated this in the fragile cancer population [15].

Here, we characterised the immune response in cancer patients with solid and haematological malignancies exposed to SARS-CoV-2 compared to unexposed cancer patients matched for age, gender, and cancer type, as well as unexposed health care workers (HCWs) from the same units that were matched for age category and gender. We aimed to investigate whether there is a protective effect or rather a higher risk for major COVID-19 complications in cancer patients. We also aimed to investigate baseline differences between solid and haematological malignancies in comparison with HCW controls without SARS-CoV-2 exposure.

## 2. Materials and Methods

### 2.1. Study Design

Ambulatory cancer patients that were scheduled for routine blood sampling attending the Multidisciplinary Oncology Unit of Antwerp University Hospital (MOCA) between 24 March 2020 and 31 May 2020, and the Oncology Unit of AZ Maria Middelares Hospital, Ghent, between 13 April 2020 and 31 May 2020, and consenting to participate (*n* = 922), were studied prospectively as part of the first COVID-19 pandemic wave in Belgium [26]. In addition, health care workers (HCWs; *n* = 92) from these units donated similar blood samples during the study period at time points 0, 1, 2, and 3 months after written informed consent. Active SARS-CoV-2 infection was diagnosed via detection of viral RNA in nose/throat swabs using the Cobas SARS-CoV-2 RT-PCR (Roche) on the automated Cobas 6800 system. Anti-SARS-CoV-2 immunoglobins (Ig) in blood were tested using three commercial tests, namely, Liaison SARS-CoV-2 S1/S2 IgG (DiaSorin, Saluggia, Italy), Alinity SARS-CoV-2 IgG (Abbott, Chicago, IL, USA), and Elecsys Anti-SARS-CoV-2 (Roche, Basel, Switzerland), as described [26]. 

**Figure 1:**
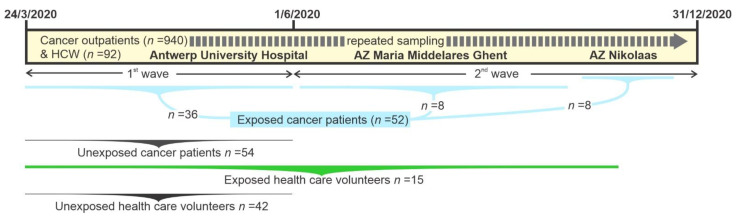
Study design. Cancer outpatients were enrolled and additional blood samples were taken with every clinically indicated blood draw. SARS-CoV-2 exposure was tested and patients were divided in groups accordingly, where unexposed patients and health care workers were matched with the exposed ones. *HCW*, health care workers.

A total of 44 cancer patients showed evidence of SARS-CoV-2 exposure with either PCR or serology and were enrolled in the current CCG study. In addition, SARS-CoV-2 PCR-positive ambulatory cancer patients attending the Multidisciplinary Oncology Unit between 1 June 2020 and 31 December 2020 as part the second COVID-19 wave were included (*n* = 10). Lastly, from a third Belgian Oncology Unit at AZ Nikolaas, Sint-Niklaas, additional SARS-CoV-2 exposed cancer (*n* = 8) patients were sampled after written informed consent between 5 November 2020 and 1 December 2020, also as part of the second COVID-19 wave. From these 62 cancer patients, 10 could not be studied as either no post-COVID-19 sample was available (*n* = 6) or the patients died due to COVID-19 consequences without a blood sample for analysis (*n* = 4). Thus, a total of 52 cancer patients were analysed for CCGs in this study. Similarly, from 92 HCWs enrolled in the first wave, 19 (20.7%) were SARS-CoV-2 positive in the first or second wave and were enrolled in the study. For 15 HCWs, a blood sample was available after SARS-CoV-2 exposure, and these were studied for blood CCGs. Besides this, unexposed cancer patients matched for age, gender, and cancer type (*n* = 54) and unexposed HCW matched for age, gender, and co-morbidity (*n* = 42) were selected as controls. The control group samples (both cancer patients and health care workers) were only included from the first wave period when the prevalence of COVID-19, and thus the chance of inadvertent exposure, was low, especially for the cancer patients who were advised to be extra vigilant (Figure 1).

Clinical data including SARS-CoV-2 related symptoms were recorded upon enrolment in the study and peak disease severity was determined according to the World Health Organization COVID-19 ordinal scale [27]. The study was approved by the ethics committee of the Antwerp University Hospital (EC number 20/13/156, internal EDGE 001070).

### 2.2. Sample Processing

For CCG immunoassays, whole blood was prospectively collected in 10 mL EDTA tubes (BD Vacutainer K2E), and plasma was prepared. Briefly, within 3 h of blood collection, samples were centrifuged at 1900× *g* for 10 min without brakes. After the first spin, plasma was transferred to a new tube and again spun at 1900× *g* for 10 min without brakes. Aliquots were flash frozen in liquid nitrogen and stored in the Biobank of Antwerp University Hospital at −80 °C until the multiplex analysis. 

### 2.3. CCG Measurements in Plasma 

CCGs were measured in EDTA plasma samples using U-plex and V-plex panels from Meso Scale Discovery (MSD, MD, USA) according to the manufacturer instructions. The following 55 CCGs were measured: brain-derived neurotrophic factor (BDNF), basic fibroblast growth factor (bFGF), C-reactive protein (CRP), cutaneous T-cell attracting chemokine (CTACK), eotaxin, erythropoietin (EPO), vascular endothelial growth factor receptor 1 (FlT-1), fractalkine, granulocyte colony stimulating factor (G-CSF), granulocyte-macrophage colony-stimulating factor (GM-CSF), macrophage colony-stimulating factor (M-CSF), interferon β (IFN-β), interferon γ (IFN-γ), interleukin (IL)-1β, IL-1 receptor antagonist (IL-1Ra), IL-2, IL-2 receptor α (IL-2Rα), IL-4, IL-5, IL-6, IL-7, IL-8, IL-9, IL-10, IL-12p40, IL-12p70, IL-13, IL-15, IL-16, IL-17A, IL-17F, IL-18, IL-21, IL-22, IL-23, IL-33, IFN-γ induced protein 10 (IP-10), monocyte chemoattractant protein (MCP)-1, MCP-2, MCP-3, macrophage inflammatory protein (MIP)-1α, MIP-1β, MIP-3α, placental growth factor (PlGF), serum amyloid A (SAA), soluble intercellular adhesion molecule 1 (sICAM-1), soluble vascular cell adhesion molecule 1 (sVCAM-1), active and total (acid activated) tumour growth factor β (TGF-β), angiopoietin receptor 1 (Tie-2), tumour necrosis factor α (TNF-α), thymic stromal lymphopoietin (TSLP), vascular endothelial growth factor (VEGF)-A, VEGF-C, and VEGF-D. 

Measurements were performed in randomized batches. Briefly, 96-well plates of the U-plex panels were coated with a capturing antibody linked to a linker for one hour. The vascular injury panel (K15198D) was washed before use. The angiogenesis panel (K15190D) was blocked with MSD blocking buffer A for one hour. All plates were then washed three times with PBS-Tween (0.05%). Samples were incubated for one hour (except for the angiogenesis and the vascular injury panels, as well as the BDNF panel where two hours of incubation were performed), after which the plates were washed three times again. Detection antibody with a sulfo-tag was added, and after another one-hour incubation step (two hours for the angiogenesis panel), plates were washed and read with MSD reading buffer on the QuickPlex SQ 120 (MSD).

### 2.4. Statistics

All data were analysed using SPSS v27, R (version R4.0.4) and MetaboAnalyst 5.0 (https://www.metaboanalyst.ca/) (accessed on 1 April 2021). CCG levels were measured in plasma samples at different timepoints for most individuals. For comparison between groups, the sample timepoint closest to the diagnosis of SARS-CoV-2 was used for the exposed individuals and the average of all samples was used for the unexposed individuals. Patient and HCW parameters were compared using the non-parametric Kruskal-Wallis test for continuous variables and Fisher’s exact test for categorical variables. Group differences in CCG profiles were explored using partial least-squares discriminant analysis (PLS-DA) with MetaboAnalyst. This analysis was performed on data normalised via autoscaling and natural log transformation. 

HCWs without SARS-CoV-2 exposure were compared with unexposed oncology patients grouped according to type of haematological and solid malignancy. One-way analysis of variance (ANOVA) was carried out to test for differences in the CCG levels between different groups followed by a post-hoc analysis with Tukey correction for multiple hypothesis testing. The test was carried out on log10-transformed values due to the non-normality of the outcome data. Reported effect sizes and fold changes were backtransformed. The significance of the test was assessed using a false discovery rate (FDR) analysis to account for the multitude of hypotheses tested, with q-values indicating the fraction of false positive associations if a given *p*-value is declared significant.

To model the change in CCG concentrations over time after SARS-CoV-2 exposure in the three groups, all timepoints after exposure were included in a linear mixed model with the logarithm of CCG concentrations as dependent variable, time as fixed effect, and individual identity as random effect to account for the non-independence of observations within the same individual. To investigate whether there was a different evolution of the CCG concentration over time between the groups, linear mixed models were fitted with time, cancer type, and their interaction as fixed effect, with the *p*-value of the interaction testing the null hypothesis that the slope was the same across all groups. In case the slope was significantly different between the groups (*p*-value for interaction <0.05), we carried out a pairwise comparison of the slopes between the HCWs and solid tumours and between HCWs and haematological malignancies. Starting from the slope estimates and their standard error, obtained through a linear mixed model of CCG versus time, a Z-test was carried out testing the null hypothesis that the slopes in the two tested groups were the same. Since two pairwise comparisons were made, a Bonferroni correction was carried out on the resulting *p*-values. 

## 3. Results

### 3.1. Patient Characteristics

A total of 52 cancer patients positive for SARS-CoV-2 PCR or serology from the first and second COVID-19 waves in Belgium were studied. Of these, 36 (69.2%) patients had a solid tumour and 16 (30.8%) had a haematological malignancy (Table 1 and Supplementary Information Table S1). Similarly, 15 HCWs who were SARS-CoV-2 positive in this study period were included in this study. Unexposed cancer patients (*n* = 54) who were matched with exposed cancer patients for age, gender and cancer type were also enrolled (Supplementary Information Table S2). Of all cancer patients, 68 patients had solid tumours and 38 haematological malignancies. Lymphomas were the most common malignancy (*n* = 17, 16%), followed by breast cancer (*n* = 16, 15%), gastric cancers (*n* = 10, 9.4%), head and neck cancers (*n* = 9, 8.5%), leukaemias (*n* = 8, 7.5%) and respiratory tract tumours (*n* = 6, 5.7%). All other malignancy types were present in less than 5% of the patients (Supplementary Information Table S2 for overview of cancer types). As healthy controls, 42 unexposed HCWs were enrolled that were group-matched with exposed HCWs for co-morbidity, age, and gender.

No significant differences were observed in patients with solid or haematological malignancies for sex and age (Table 1). When comparing both cancer types with HCWs, there were significantly fewer males in the HCW cohorts (12.3% males) compared to both cancer groups (solid unexposed/exposed 53.1%/41.7%; haematological unexposed/exposed 40.6%/43.8%). Additionally, HCWs were significantly younger (average (Av) 36 years, *p* < 0.001) than both groups of cancer patients (solid cancer Av 59.6 years; haematological malignancy Av 55.4 years). BMI was not significantly different between groups (solid cancers Av 25.6; haematological malignancies Av 24.3; HCWs Av 24.1). None of the HCWs were diabetic compared to 8 (11.8%) in the solid and 1 (2.6%) in the haematological malignancy patient groups. Furthermore, there were fewer infections other than SARS-CoV-2 in HCWs (3.5% overall) compared to solid tumour patients (16.2% overall) and haematological malignancy patients (28.9% overall) (Supplementary Information Table S1). Nine patients in the haematological malignancy group received bone marrow transplantation (*n* = 9). Cancer patients received chemotherapy (*n* = 67), antihormonal therapy (*n* = 6), or targeted therapy such as monoclonal antibodies, proteasome inhibitors, signal transduction inhibitors, and angiogenesis inhibitors (*n* = 37).

### 3.2. No Significant Difference in Disease Severity between SARS-CoV-2 Exposed Cancer Patients and Exposed Health Care Workers

We studied whether SARS-CoV-2 exposed cancer patients or HCWs in our cohorts had a different disease severity. Disease severity for the patients enrolled for the CCG study was graded as asymptomatic, mild, moderate, severe, and critical [27] (Table 1). For this analysis, we also included the 10 additional patients that were excluded for the CCG study due to the lack of plasma samples. While no significant difference was observed for any of the severity groups between HCWs and the cancer groups, 89.5% of HCWs and 71.1% and 82.4% of the solid and haematological malignancy group, respectively, remained asymptomatic or showed mild to moderate symptoms (Supplementary Information Figure S1). Although a slightly higher disease severity is observed in the solid cancer cohort, this difference was not significant (Fisher’s exact test). Bearing in mind that cancer patients were approximately 20 years older, and gender balanced (males 49.1%) while HCWs were mostly females (87.7%), these data suggest that cancer patients are not significantly different for disease severity in the cohort studied here.

### 3.3. In Absence of SARS-CoV-2 Exposure, CCG Profiles in Solid and Haematological Malignancies Are Not Inherently Different from Each Other, but Different from Unexposed Healthy Controls

**Figure 2:**
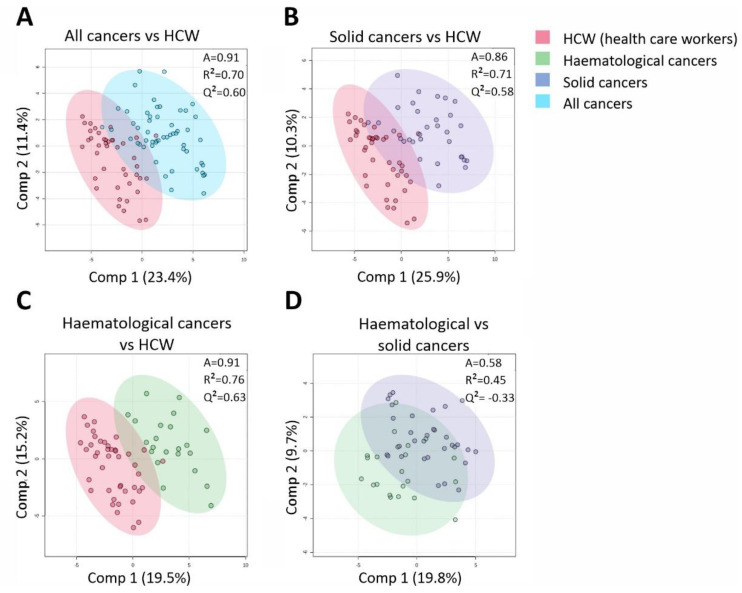
Cluster analysis of CCGs in unexposed individuals with partial least-squares discriminant analysis (PLS-DA) reveals larger differences between health care workers (HCWs) and cancer as a group or individually as solid and haematological groups, than between the two cancer groups. (**A**) The comparison of HCWs with the two different cancer types pooled. (**B**) The comparison of the HCWs with patients with solid tumours (**C**) and with haematological malignancies (**D**) Comparison of haematological versus solid cancer patients. A, accuracy.

We analysed plasma samples of unexposed cancer patients with either solid or haematological malignancies as well as unexposed HCWs for 55 CCGs (Figure 2). Partial least squares discriminant analysis (PLS-DA) identified a clear clustering of the HCWs and combined group of cancer patients (accuracy = 91%, R^2^ = 0.76, Q^2^ = 0.63 for 2 components; Figure 2A). High classification accuracies were also achieved between the unexposed HCW group and unexposed solid tumours (accuracy = 86%, R^2^ = 0.71, Q^2^ = 0.58; Figure 2B) and between the unexposed HCWs and haematological malignancies (Accuracy 91%, R^2^ = 0.76, Q^2^ = 0.63; Figure 2C). However, we did not find a clear clustering of the CCG profiles between haematological and solid malignancies (Q^2^ = −0.33 for 2 components; Figure 2D). Further analysing the individual cytokine differences between solid and haematological malignancies with a one-way analysis of variance (ANOVA), we found a statistically significant increase in only 3 CCGs in patients with solid tumours compared to those with haematological malignancies: BDNF (4.1-fold, *p* = 0.004), VEGF-C (2.2-fold, *p* = 0.019), and SAA (2.2-fold, *p* = 0.016). These data suggest that while the baseline CCG profiles in unexposed patients with solid or haematological malignancies are significantly different from the unexposed HCWs, CCG profiles of unexposed patients with either solid or haematological malignancies are not grossly distinct.

### 3.4. A Broad Group of Inflammatory Markers and Growth Factors Distinguishes Unexposed Solid or Haematological Malignancies from Healthy Controls

**Figure 3:**
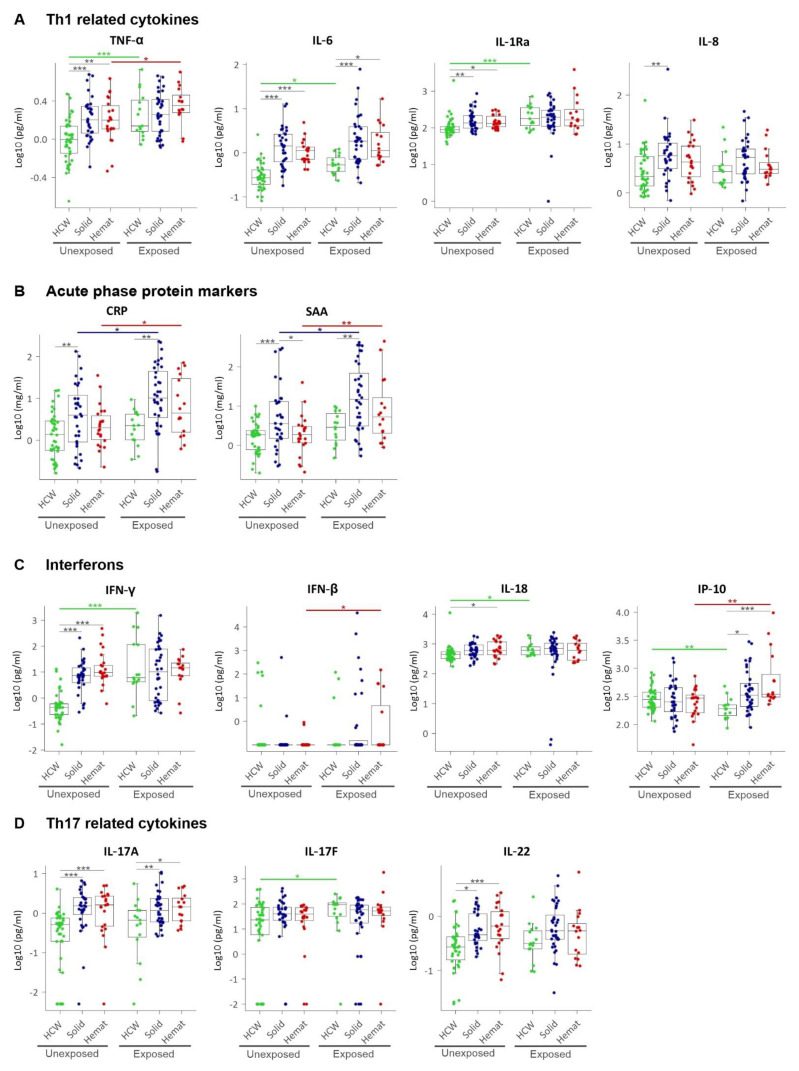
Plasma levels of pro-inflammatory CCGs as (**A**) Th1 related cytokines, (**B**) acute phase proteins, (**C**) interferons and interferon-related cytokines, and (**D**) Th17 related cytokines. HCW, health care workers (green); solid, patients with solid tumours (blue); hemat, patients with haematological malignancies (red). Each dot represents the sample closest to exposure in the case of exposed patients, and an average of multiple timepoints, when available, for unexposed patients. * *p* < 0.05, ** *p* < 0.01, *** *p* < 0.001.

**Figure 4:**
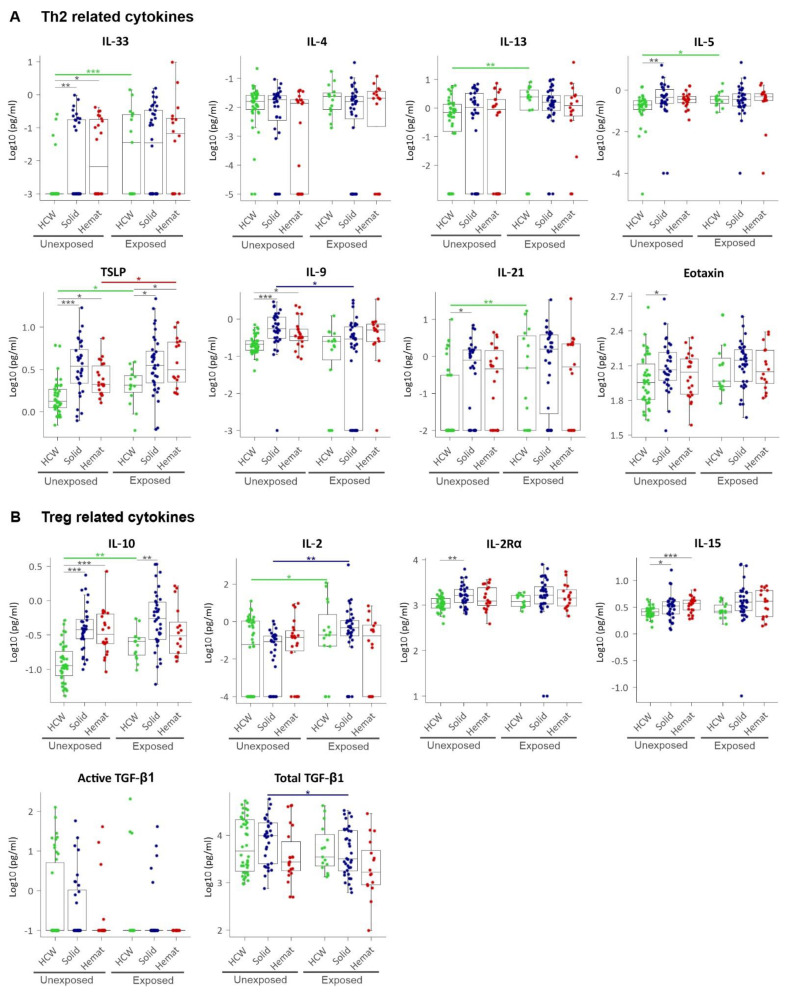
Plasma levels of (**A**) Th2 related (anti-inflammatory) cytokines and (**B**) Treg-related (immunomodulatory) cytokines. HCW, health care workers (green); solid, patients with solid tumours (blue); hemat, patients with haematological malignancies (red). Each dot represents the sample closest to exposure in the case of exposed patients, and an average of multiple timepoints, when available, for unexposed patients. * *p* < 0.05, ** *p* < 0.01, *** *p* < 0.001.

**Figure 5:**
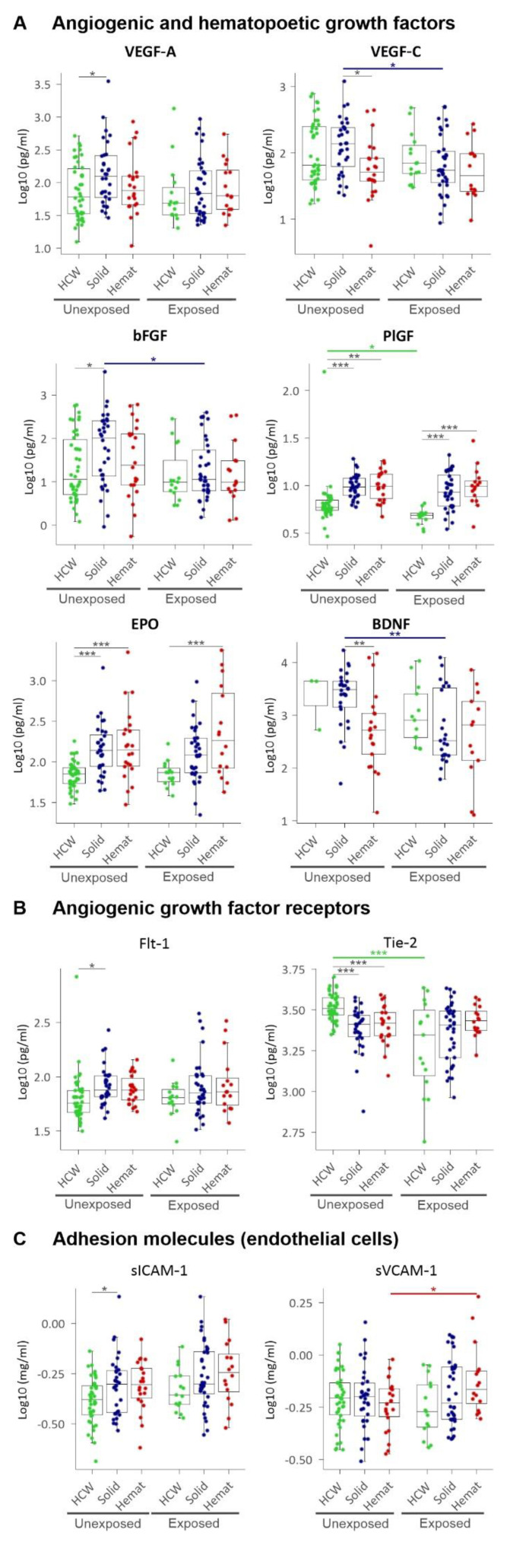
Plasma levels of (**A**) angiogenic and haematopoietic growth factors, (**B**) angiogenic growth factor receptors, and (**C**) adhesion molecules expressed by endothelial cells. HCW, health care workers (green); solid, patients with solid tumours (blue); hemat, patients with haematological malignancies (red). Each dot represents the sample closest to exposure in the case of exposed patients and an average of multiple timepoints, when available, for unexposed patients. * *p* < 0.05, ** *p* < 0.01, *** *p* < 0.001.

**Figure 6:**
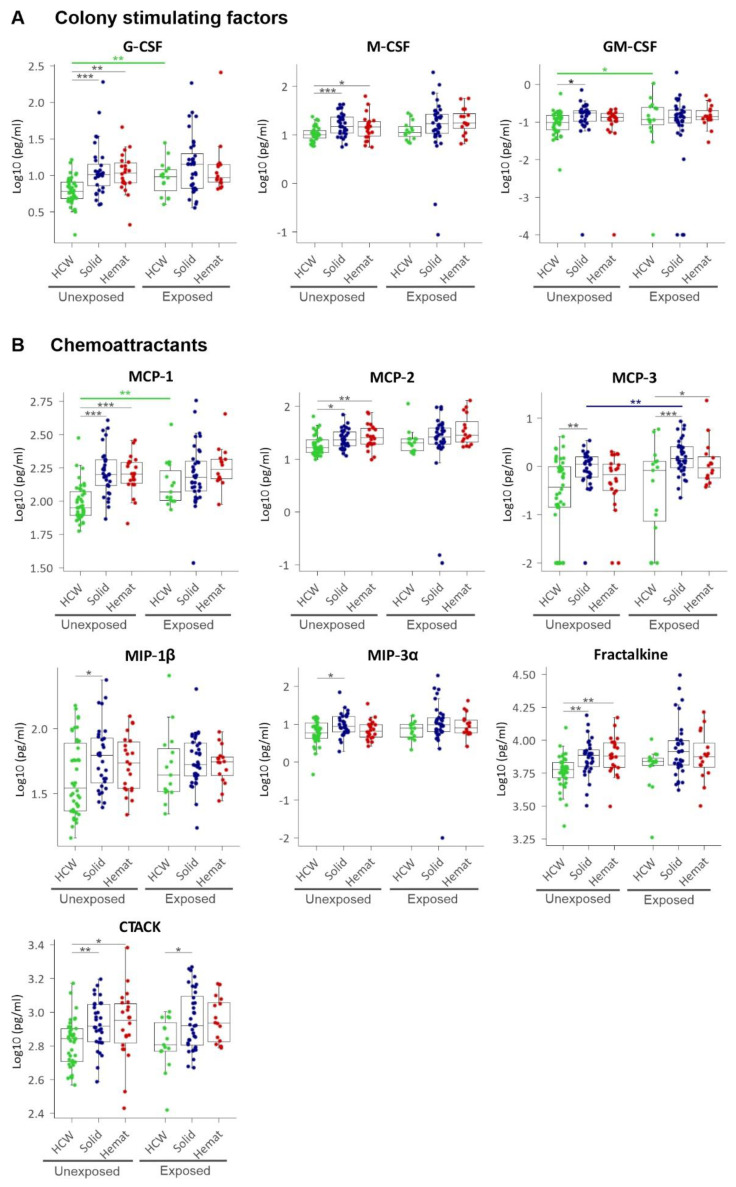
Plasma levels of (**A**) colony stimulating factors (CSFs) and (**B**) monocyte chemoattracting proteins (MCPs), macrophage inflammatory proteins (MIPs) and other chemokines. HCW, health care workers (green); solid, patients with solid tumours (blue); hemat, patients with haematological malignancies (red). Each dot represents the sample closest to exposure in the case of exposed patients and an average of multiple timepoints, when available, for unexposed patients. * *p* < 0.05, ** *p* < 0.01, *** *p* < 0.001.

Next, we studied the basis of the discriminant CCG profiles between both groups of unexposed cancer patients and unexposed HCWs using ANOVA. These results are presented in Figure 3, Figure 4, Figure 5, Figure 6, Supplementary Information Figures S2 and S3A, and Supplementary Information Table S3. First, consistent with the role of inflammation in cancer development and progression [2], we observed a significant upregulation of Th1 pro-inflammatory cytokines in both solid and haematological malignancy patients compared to HCWs, including TNF-α, IL-6, and IL-1Ra (Figure 3A). A significant upregulation of neutrophilic chemokine IL-8 and acute phase proteins CRP and SAA was also observed for solid tumours, but not for haematological malignancy patients (Figure 3B). Regarding the interferon family, while IFN-β and IP-10 were not significantly altered, IFN-γ was significantly increased in both solid and haematological malignancy patients, and IL-18 (also called IFN-γ-inducing factor) only in the later cancer group (Figure 3C). Similarly, pro-inflammatory Th17-related cytokines, IL-17A and IL-22 were significantly upregulated in both solid and haematological malignancy patients compared to HCWs (Figure 3D).

We further studied the Th2 family of cytokines, which besides being anti-inflammatory are promotors of tumour cell growth and invasion via enhancing pro-tumour properties of macrophages [28]. We observed a significant upregulation of IL-33, TSLP, and IL-9 for both solid and haematological malignancy patients compared to HCWs, and for IL-5, eotaxin, and IL-21 in only solid tumour patients (Figure 4A). However, no significant differences in the major Th2 cytokine IL-4 and IL-13 levels were observed for either of the cancer groups compared to HCWs. For immunomodulatory cytokines, IL-10 and IL-15 were found to be significantly upregulated in both solid and haematological malignancy patients compared to HCWs, while another Treg-associated cytokine, IL-2Rα, was significantly upregulated only in solid tumour patients (Figure 4B).

We also addressed angiogenic growth factors (i.e., VEGF family, bFGF, PlGF), their receptors (Flt-1, Tie-2), haematopoietic stimulators (EPO, IL-7), as well as adhesion molecules expressed by endothelial cells (sICAM, sVCAM). Besides this, we also studied BDNF, a newly identified mediator of angiogenesis that acts through stimulating VEGF [29]. While angiogenesis plays a critical role in the progression of solid tumours, it is increasingly recognized that haematological malignancies also depend on the induction of new blood vessel formation [14]. Consistent with these data, we showed that while VEGF-A and its receptor Flt-1, bFGF, and ICAM were significantly upregulated for solid tumours, PlGF and EPO were significantly upregulated for both solid and haematological malignancy patients compared to HCWs. Interestingly, angiopoietin receptor Tie-2 was significantly 30–35% downregulated in both solid and haematological malignancy patients compared to HCWs (Figure 5).

Lastly, we addressed the question of whether colony stimulating factors and chemokines were distinctly altered in solid or haematological malignancies. We first showed that colony stimulating factors for neutrophils (G-CSF) and macrophages (M-CSF) were significantly upregulated in both solid and haematological malignancy patients, compared to HCW controls, while GM-CSF, a broader stimulant for all granulocytes and monocytes, was only upregulated in solid cancer patients (Figure 6A). Concerning chemokines, we showed that monocyte chemotactic proteins MCP-1 and -2—considered as principal chemokines involved in the recruitment of monocytes/macrophages and activated lymphocytes—were significantly upregulated in both solid and haematological malignancies, while closely related MCP-3 and MIP-1β/CCL4, implicated in the chemotaxis of dendritic cells and eosinophils, were only upregulated in solid tumours but not haematological malignancies. Similarly, CCL20/MIP-3α, a chemotactic factor for lymphocytes, was also upregulated only in solid tumours, while CCL27/CTACK was upregulated for both solid and haematological malignancies. Lastly, CX3CL1/fractalkine, a chemokine abundantly expressed by activated endothelium and promoting strong adhesion of leukocytes to endothelial cells, which is also involved in thrombosis, was significantly upregulated by up to 30% in both solid and haematological malignancy patients compared to HCWs (Figure 6B). These data suggest gross alterations in CCG profiles of solid or haematological malignancy patients, with one important mediator, Tie-2, remarkably downregulated.

### 3.5. SARS-CoV-2 Exposure Elicits an Expected Increase in CCG Levels in HCWs

Next, we studied how CCG levels in plasma of SARS-CoV-2 altered in exposed HCWs compared to those who were unexposed. Here, significantly higher levels of the Th1 cytokines and acute phase proteins—TNF-α (1.4-fold), IL-1Ra (2.0-fold), IL-6 (1.2-fold), and SAA (1.5-fold)—were observed in exposed HCWs compared to unexposed HCWs (Figure 3A,B; Supplementary Information Figure S3B, Supplementary Information Table S4A). For the interferon family, IFN-γ was significantly increased (11.8-fold) whereas IP-10 was significantly decreased (1.5-fold) (Figure 3C) along with the proinflammatory IL-17F (3-fold increased) (Figure 3D). Interestingly, most Th2 cytokines were also significantly increased in HCWs after exposure to SARS-CoV-2 and included IL-33 (1.2-fold), IL-5 (1.2-fold), IL-13 (1.6-fold), TSLP (1.2-fold) and IL-21 (1.8-fold) (Figure 4A). Immunomodulatory IL-10 was also found to be slightly but significantly increased in exposed HCWs (1.1-fold) along with Treg-associated IL-2 (1.9-fold; Figure 4B). While exposed HCWs showed a decrease in angiogenic growth factor PlGF (1.3-fold) and angiopoietin receptor Tie-2 (1.7-fold) (Figure 5), an increase was found for G-CSF (1.4-fold), GM-CSF (1.1-fold), and monocytes/macrophages chemoattractant MCP-1 (1.4-fold) (Figure 6). Although we predominantly studied post-acute timepoints and the patients were mostly in milder categories, these data are in agreement with COVID-19 where similar alterations in CCGs are observed in severe COVID-19 at acute timepoints [21,22,23,24,25,30,31,32,33].

### 3.6. SARS-CoV-2 Exposure Causes Noteworthy Alterations in CCG Profiles of Solid and Haematological Malignancy Patients

More central to the main hypothesis of the paper, we further studied whether SARS-CoV-2 exposure in cancer patients elevates CCG linked to cancer progression. Despite elevated levels of several inflammatory mediators already occurring in cancer patients in comparison to non-cancer controls, an addition of 7 CCGs were additionally found to be significantly altered in cancer patients exposed to SARS-CoV-2. Specifically, in patients with solid tumours, besides a significant upregulation of the inflammatory markers CRP (2.1-fold) and SAA (2.5-fold), only immune cell activators IL-2 (1.9-fold) and MCP-3 (1.3-fold) were elevated (Figure 3, Figure 4, Figure 5 and Figure 6, Supplementary Information Figure S3B, Supplementary Information Table S4B). In contrast, 5 CCGs showed a significant reduction in solid malignancy patients exposed to SARS-CoV-2, namely, angiogenesis growth factors VEGF-C (1.9-fold), bFGF (2.9-fold), and BDNF (3.7-fold), as well as IL-9 (1.2-fold) and total TGF-β (1.8-fold). 

Similar to patients with solid tumours, individuals with haematological tumours exposed to SARS-CoV-2 showed a significant increase in the inflammatory markers CRP (2.4-fold) and SAA (3.1-fold). Furthermore, there was an increase in TNF-α (1.3-fold), IP-10 (2.5-fold), TSLP (1.4-fold), VCAM-1 (1.1-fold) and antiviral IFN-β (2.7-fold) (Figure 3, Figure 4, Figure 5 and Figure 6, Supplementary Information Table S4C).

### 3.7. Longitudinal Analysis Shows Persistence of CCG Alterations in SARS-CoV-2 Exposed Cancer Patients

Because several CCGs are involved in tumour progression, we studied the temporal evolution of the 14 CCGs for which we showed a significant increase or decrease for the exposed cancer groups. Samples from multiple timepoints collected over three months were available for the exposed cancer and HCW groups and were analysed. CCG levels were log transformed and entered in a linear mixed model to test whether the 14 CCGs for the cancer and HCW groups significantly changed over time and whether the rate of change for solid cancer or haematological malignancy groups significantly differed from that of the HCW group.

**Figure 7:**
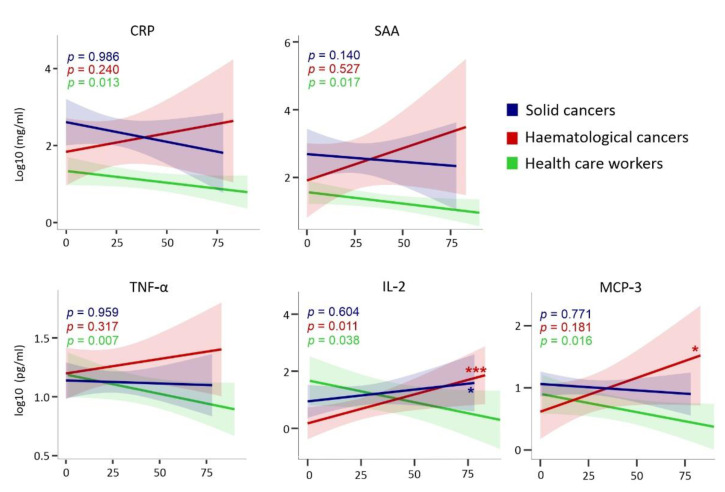

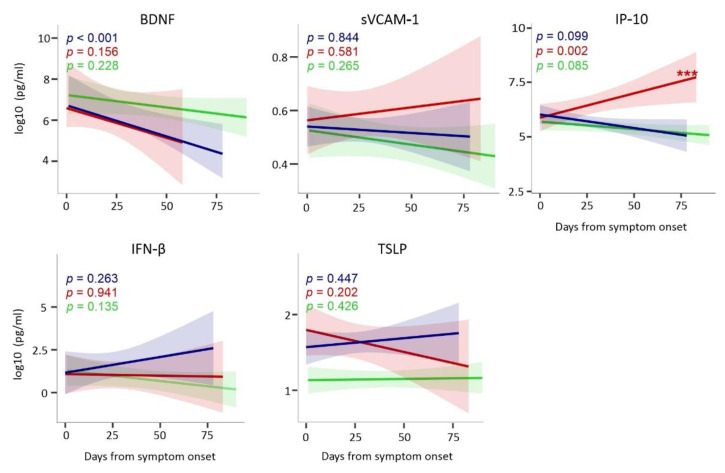
Temporal evolution of CCGs altered in exposed cancer patients. HCW, health care workers (green); solid, patients with solid tumours (blue); hemat, patients with haematological malignancies (red). Time is represented as days since symptom onset (day 0). *p*-values in the graph refer to significance of the slope from the 3 separate regression lines. The asterisks represent the significance of the pairwise comparison between the slope (*p*-value for interaction) in haematological malignancy versus HCW (red) and solid malignancy versus HCW (blue). *p < 0.05, *** *p* < 0.001.

We first showed that the inflammatory mediators CRP and SAA significantly declined in HCWs after SARS-CoV-2 exposure. A non-significant declining trend was also observed for solid tumours, but not for the haematological malignancy group suggesting a persistence of pro-inflammatory state in cancer patients, especially haematological malignancy patients, exposed to SARS-CoV-2 (Figure 7). A persistence of CCGs such as TNF-α, IL-2, and MCP-3 was also observed for exposed cancer patients but not for the healthy control group. For the haematological malignancy patient group, levels of IL-2 and MCP-3 even temporarily increased in the three-month follow-up and was significantly different from the declining levels noted for healthy controls. A significant temporal increase in IP-10 was also observed for haematological malignancy patients compared to a non-significant declining trend for healthy controls. These data suggest that several CCGs shown to be significantly increased in the exposed cancer patients compared to unexposed cancer patients are in fact long-lasting changes that could persist for at least 3 months after SARS-CoV-2 exposure.

## 4. Discussion

In a highly diverse set of cancer types representing the most common types of solid as well as haematological malignancies, we identified distinct immunological profiles of CCGs in these two types of cancers. While 19 CCGs were elevated in both solid and haematological malignancy patients, a further 15 CCGs were uniquely upregulated in solid tumours while only one CCG (IL-18) was uniquely upregulated in haematological malignancies. In addition, only one CCG (angiopoietin receptor Tie-2) was also significantly downregulated (30–35%) in cancer patients and this was observed for both solid and haematological malignancy. Besides a function in angiogenesis, Tie-2 also controls cellular adhesion and invasion and therefore metastatic behaviour, and a downregulation of Tie-2 has been reported in squamous cell carcinomas in cell lines and tissue [34].

Alluding further on the differences between solid and haematological malignancies, we identified 3 CCGs that passed a stringent significance threshold for multiple comparison statistics for 55 CCGs. These 3 CCGs were SAA, VEGF-C, and BDNF and were found to be higher in solid tumour patients. While a critical role of inflammation in progression of haematological malignancy including myeloid malignancies is now recognized [35], an increased acute phase marker protein SAA in solid tumours compared to haematological malignancy patients suggests that inflammation is more central to solid tumours. Similarly, an association of VEGF-C and BDNF with solid tumours is not surprising since tumour microenvironment remodelling with vascular growth aids in the growth and maintenance of solid tumours while being less pronounced in haematological malignancies [13,29,36].

In this background biology of CCG alterations in cancer patients, we further studied how SARS-CoV-2 exposure alters the CCGs in cancer patients. This was conducted as part of a prospective surveillance study covering the first and second COVID-19 wave in Belgium. Most of the studied patients were asymptomatic or had mild disease while only a minority required hospitalization. Keeping that in mind, we first describe a significant upregulation in levels of 17 CCGs in HCWs exposed to SARS-CoV-2 compared to unexposed HCWs while 3 CCGs were significantly downregulated (Tie-2, PlGF, and IP-10). However, likely because CCGs were already highly elevated in the unexposed cancer groups, the effect on CCG alteration in SARS-CoV-2-exposed solid and haematological malignancy patients was less pronounced compared to the respective unexposed cancer groups. Only SAA and CRP were commonly elevated in exposed solid and haematological malignancies, and these represent the two most common acute phase protein markers employed in COVID-19 as well as in other inflammatory and infectious conditions and importantly suggest a prolonged pro-inflammatory state in exposed cancer patients [37]. Moreover, SAA has been previously shown to elicit immune evasion by the tumour through the reprogramming of macrophages to the pro-tumour M2 type and downregulation of anti-tumour responses [38].

In addition, we reported 2 CCGs (MCP-3 and IL-2) that were uniquely elevated and 5 CCGs (IL-9, VEGF-C, bFGF, BDNF and total TGF-β) that were uniquely downregulated in the solid tumour group exposed to SARS-CoV-2 compared to unexposed. While decreased VEGF-C, bFGF, and BDNF could dysregulate angiogenesis in solid tumours, a decreased IL-9 and elevated MCP-3, SAA, and IL-2 might help to accelerate progression of solid tumours. We also showed that IL-2 was amongst the selected cytokines that remained significantly elevated in the SARS-CoV-2 exposed solid tumour group over a 3-month study period. IL-2 is a major recruiter of T-helper cells, and also promotes self-tolerance via expansion of regulatory T cells [39]. Similarly, MCP-3 has been shown to help support the tumour microenvironment through the recruitment of tumour-associated lymphocytes and is also associated with infiltration of tumour-associated macrophages that aid immune evasion [40,41]. 

Conversely, patients with haematological malignancies showed a unique elevation for 5 CCGs (TNF-α, IFN-β, TSLP, soluble VCAM-1, and IP-10). While TNF-α, IFN-β, TSLP, SAA, and sVCAM-1 are known to promote growth or enable immune evasion in haematological tumours [5,38,42,43,44], the unique elevation of IP-10 in haematological malignancies is also noteworthy as it promotes angiogenesis and metastasis in solid tumours and has also been found in increased levels in chronic myelomonocytic leukaemia patients [45,46]. Recent evidence has also emerged that highlights intriguing parallels between inflammatory pathways and aberrant immune cell crosstalk in metastasis formation and the role that primary tumours play in hijacking these interactions to enhance their metastatic potential [47]. Taken together, these data suggest that CCG profiles in haematological patients seem to be altered towards promotion of cancer progression after SARS-CoV-2 exposure. This is even more concerning as not only do levels of CCGs such as MCP-3 and TNF-α show no decline until at least 3 months after SARS-CoV-2 exposure, but levels of IL-2 and IP-10 even increase during this period. A recent study has also showed sustained immune dysregulation in haematological cancer patients displaying heterogeneous humoral responses and an exhausted T cell phenotype up to three months after in SARS-CoV-2 exposure [15]. Together these data provide evidence for the need for increased vigilance in clinical follow up for any sequelae of faster cancer progression after SARS-CoV-2 infection in patients with haematological malignancies, as has been speculated recently [48,49,50].

On the other hand, the question remains whether elevated baseline cytokine levels in cancer patients might offer protection in the initial stages of SARS-CoV-2 infection. While some studies have not reported increased COVID-19 severity in cancer patients [19], those that did have only considered hospitalized patients [16]. Many of the elevated cytokines in unexposed cancer patients in our study, especially with solid tumours, include pro-inflammatory Th1-related cytokines, which are classically targeted against bacteria and viruses. In our cancer cohort, SARS-CoV-2 exposure was only reported in approximately 4% of cancer patients compared to 3.1–6.9% in the overall Belgian population in the same time interval [51] and approximately 12% in the HCWs [26]. It is likely this can be explained by better self-protection of cancer patients. While cancer patients had more SARS-CoV-2-related mortality and severe/critically illness, 71% of the exposed solid cancer patients remained asymptomatic or mild to moderately ill, which was not significantly different from non-severe illness showed by HCWs (89.5%). This is noteworthy as the HCWs in our study were, on average, 24 years younger and had fewer co-morbidities than cancer patients. It is tempting to speculate that high baseline levels of innate pro-inflammatory cytokines in cancer patients might be beneficial to rapidly deal with low-level exposure to SARS-CoV-2. However, it seems that once the balance of the “primed” immune system is disrupted, COVID-19 can be more severe and lethal in a subset of cancer patients.

As limitations, this study is a case-control study and prospective data collection, especially for patients who were not positive for SARS-CoV-2, was not possible. Secondly, although older healthy controls were enrolled for this study, this group remained younger and had fewer co-morbidities compared to the cancer groups. Lastly, as the study was conducted in an oncology unit setting, cancer patients that were immediately transferred to the COVID-19 wards and had succumbed to the infection, were not included. 

## 5. Conclusions

We show here that cancer patients have intrinsically high levels of inflammatory cytokines/chemokines as well as angiogenic and other growth factors, which escalate significantly after SARS-CoV-2 infection, especially in haematological malignancy patients. Moreover, while cytokine profiles in patients with solid tumours stabilised over time, patients with haematological malignancies showed a sustained dysregulated immune response persisting for up to 3 months during the study period. As several of the cytokines/chemokines and growth factors studied here are also tumour-promoting factors, our data calls for increased vigilance in patients with haematological malignancy with SARS-COV-2 infection as a part of long COVID-19 surveillance.

## Figures and Tables

**Table 1:** Patient characteristics *.

	Solid Cancer (*n* = 68)		Haematological Cancer (*n* = 38)		HCWs (*n* = 57)	
	% or Mean (SD)		% or Mean (SD)		% or Mean (SD)	
	Exposed ^a^	Unexp ^b^	Exposed ^a^	Unexp ^b^	Exposed ^a^	Unexp ^b^
SARS-CoV-2 status	*n* = 36	*n* = 32	*n* = 16	*n* = 22	*n* = 15	*n* = 42
Asymptomatic	22.2%	NA	31.3%	NA	40.0%	NA
Mild	52.8%	NA	37.5%	NA	46.7%	NA
Moderate	8.3%	NA	12.5%	NA	0%	NA
Severe	16.7%	NA	12.5%	NA	13.3%	NA
Critical	0%	NA	6.3%	NA	0%	NA
Male sex	41.7%	53.1%	43.8%	40.6%	13.3%	11.9%
Age, mean (SD)	58.8 (13.6)	60.5 (12.7)	57.9 (13.9)	53.6 (20.4)	37.5 (11.4)	35.5 (10.7)
BMI, mean (SD)	25.7 (6.1)	25.6 (6.4)	24.6 (6.9)	24.1 (4.0)	22.9 (0.0)	24.2 (3.7)
Recent chemotherapy	61.1%	62.5%	68.8%	46.9%	0%	0%
Radiotherapy	22.2%	15.6%	18.8%	9.4%	0%	0%
Recent targeted therapy	33.3%	28.1%	50%	28.1%	0%	0%
Antihormonal treatment	5.6%	9.4%	6.3%	0%	0%	0%
Transplant	0%	0%	25.0%	15.6%	0%	0%

HCWs: health care workers.SD: standard deviation. ^a^ All SARS-CoV-2 exposed individuals were included for CCG analysis. ^b^ Unexposed individuals were group-matched to exposed individuals based on age, gender, and cancer type in the case of cancer patients. NA: not applicable. * Additional patient comorbidities are represented in Supplementary Table S1.

## Data Availability

The data that support the findings of this study are available from the corresponding author upon reasonable request.

## Supplementary Materials

The following are available online at https://www.mdpi.com/article/10.3390/cancers13225718/s1, Figure S1: Proportion of patients according to severity in solid and haematological malignancies as well as HCWs, Figure S2: Plasma levels of additional CCGs, Figure S3: Summarising figure showing cytokines significantly increased and decreased, Table S1: Patient characteristics, Table S2: Overview of all patients with information on cancer types, topography, morphology and behaviour of the malignancy, Table S3: Alterations in CCGs for unexposed solid and haematological malignancy patients compared to unexposed healthcare workers, Table S4: Alterations in CCGs levels in SARS-CoV-2-exposed individuals compared to unexposed individuals.

## References

[1] Mantovani A., Allavena P., Sica A., Balkwill F. (2008). Cancer-related inflammation. Nature.

[2] Grivennikov S.I., Greten F.R., Karin M. (2010). Immunity, Inflammation, and Cancer. Cell.

[3] Lan T., Chen L., Wei X. (2021). Inflammatory Cytokines in Cancer: Comprehensive Understanding and Clinical Progress in Gene Therapy. Cells.

[4] Johnson D.E., O’Keefe R.A., Grandis J.R. (2018). Targeting the IL-6/JAK/STAT3 signalling axis in cancer. Nat. Rev. Clin. Oncol..

[5] Balkwill F. (2006). TNF-alpha in promotion and progression of cancer. Cancer Metastasis Rev..

[6] Do H.T.T., Lee C.H., Cho J. (2020). Chemokines and their Receptors: Multifaceted Roles in Cancer Progression and Potential Value as Cancer Prognostic Markers. Cancers.

[7] Tokunaga R., Nakagawa S., Sakamoto Y., Nakamura K., Naseem M., Izumi D., Kosumi K., Taki K., Higashi T., Miyata T. (2020). 12-Chemokine signature, a predictor of tumor recurrence in colorectal cancer. Int. J. Cancer..

[8] Chen Z.Y., He W.Z., Peng L.X., Jia W.H., Guo R.P., Xia L.P., Qian C.N. (2015). A prognostic classifier consisting of 17 circulating cytokines is a novel predictor of overall survival for metastatic colorectal cancer patients. Int. J. Cancer. J. Int. Cancer.

[9] Capone F., Guerriero E., Sorice A., Colonna G., Ciliberto G., Costantini S. (2016). Serum Cytokinome Profile Evaluation: A Tool to Define New Diagnostic and Prognostic Markers of Cancer Using Multiplexed Bead-Based Immunoassays. Mediat. Inflamm..

[10] Shiels M.S., Pfeiffer R.M., Hildesheim A., Engels E.A., Kemp T.J., Park J.-H., Katki H.A., Koshiol J., Shelton G., Caporaso N.E. (2013). Circulating Inflammation Markers and Prospective Risk for Lung Cancer. JNCI J. Natl. Cancer Inst..

[11] Arfsten H., Cho A., Freitag C., Raderer M., Goliasch G., Bartko P.E., Wurm R., Strunk G., Gisslinger H., Marosi C. (2019). GDF-15 in solid vs non-solid treatment-naïve malignancies. Eur. J. Clin. Investig.

[12] Weinstein P.S., Skinner M., Sipe J.D., Lokich J.J., Zamcheck N., Cohen A.S. (1984). Acute-phase proteins or tumour markers: The role of SAA, SAP, CRP and CEA as indicators of metastasis in a broad spectrum of neoplastic diseases. Scand. J. Immunol..

[13] Carmeliet P. (2005). VEGF as a key mediator of angiogenesis in cancer. Oncology.

[14] Medinger M., Passweg J. (2014). Role of tumour angiogenesis in haematological malignancies. Swiss Med. Wkly..

[15] Abdul-Jawad S., Baù L., Alaguthurai T., Del Molino Del Barrio I., Laing A.G., Hayday T.S., Monin L., Muñoz-Ruiz M., McDonald L., Francos Quijorna I. (2021). Acute Immune Signatures and Their Legacies in Severe Acute Respiratory Syndrome Coronavirus-2 Infected Cancer Patients. Cancer Cell.

[16] Liang W., Guan W., Chen R., Wang W., Li J., Xu K., Li C., Ai Q., Lu W., Liang H. (2020). Cancer patients in SARS-CoV-2 infection: A nationwide analysis in China. Lancet Oncol..

[17] Dai M., Liu D., Liu M., Zhou F., Li G., Chen Z., Zhang Z., You H., Wu M., Zheng Q. (2020). Patients with Cancer Appear More Vulnerable to SARS-CoV-2: A Multicenter Study during the COVID-19 Outbreak. Cancer Discov..

[18] Tian Y., Qiu X., Wang C., Zhao J., Jiang X., Niu W., Huang J., Zhang F. (2021). Cancer associates with risk and severe events of COVID-19: A systematic review and meta-analysis. Int. J. Cancer.

[19] Lee L.Y., Cazier J.B., Angelis V., Arnold R., Bisht V., Campton N.A., Chackathayil J., Cheng V.W., Curley H.M., Fittall M.W. (2020). COVID-19 mortality in patients with cancer on chemotherapy or other anticancer treatments: A prospective cohort study. Lancet.

[20] van Dam P.A., Huizing M., Mestach G., Dierckxsens S., Tjalma W., Trinh X.B., Papadimitriou K., Altintas S., Vermorken J., Vulsteke C. (2020). SARS-CoV-2 and cancer: Are they really partners in crime?. Cancer Treat. Rev..

[21] Lucas C., Wong P., Klein J., Castro T.B.R., Silva J., Sundaram M., Ellingson M.K., Mao T., Oh J.E., Israelow B. (2020). Longitudinal analyses reveal immunological misfiring in severe COVID-19. Nature.

[22] Huang C., Wang Y., Li X., Ren L., Zhao J., Hu Y., Zhang L., Fan G., Xu J., Gu X. (2020). Clinical features of patients infected with 2019 novel coronavirus in Wuhan, China. Lancet.

[23] Del Valle D.M., Kim-Schulze S., Huang H.H., Beckmann N.D., Nirenberg S., Wang B., Lavin Y., Swartz T.H., Madduri D., Stock A. (2020). An inflammatory cytokine signature predicts COVID-19 severity and survival. Nat. Med..

[24] Abers M.S., Delmonte O.M., Ricotta E.E., Fintzi J., Fink D.L., de Jesus A.A.A., Zarember K.A., Alehashemi S., Oikonomou V., Desai J.V. (2021). An immune-based biomarker signature is associated with mortality in COVID-19 patients. JCI Insight.

[25] Young B.E., Ong S.W.X., Ng L.F.P., Anderson D.E., Chia W.N., Chia P.Y., Ang L.W., Mak T.M., Kalimuddin S., Chai L.Y.A. (2020). Viral dynamics and immune correlates of COVID-19 disease severity. Clin. Infect. Dis. Off. Publ. Infect. Dis. Soc. Am..

[26] van Dam P., Huizing M., Roelant E., Hotterbeekx A., De Winter F.H.R., Kumar-Singh S., Moons P., Amajoud Z., Vulsteke C., Croes L. (2021). Immunoglobin G/total antibody testing for SARS-CoV-2: A prospective cohort study of ambulatory patients and health care workers in two Belgian oncology units comparing three commercial tests. Eur. J. Cancer.

[27] WHO Working Group on the Clinical Characterisation and Management of COVID-19 Infection (2020). A minimal common outcome measure set for COVID-19 clinical research. Lancet. Infect. Dis..

[28] DeNardo D.G., Barreto J.B., Andreu P., Vasquez L., Tawfik D., Kolhatkar N., Coussens L.M. (2009). CD4(+) T cells regulate pulmonary metastasis of mammary carcinomas by enhancing protumor properties of macrophages. Cancer Cell.

[29] Kermani P., Hempstead B. (2007). Brain-derived neurotrophic factor: A newly described mediator of angiogenesis. Trends Cardiovasc. Med..

[30] Thwaites R.S., Sanchez Sevilla Uruchurtu A., Siggins M.K., Liew F., Russell C.D., Moore S.C., Fairfield C., Carter E., Abrams S., Short C.E. (2021). Inflammatory profiles across the spectrum of disease reveal a distinct role for GM-CSF in severe COVID-19. Sci. Immunol..

[31] Burke H., Freeman A., Cellura D.C., Stuart B.L., Brendish N.J., Poole S., Borca F., Phan H.T.T., Sheard N., Williams S. (2020). Inflammatory phenotyping predicts clinical outcome in COVID-19. Respir. Res..

[32] Yang Y., Shen C., Li J., Yuan J., Wei J., Huang F., Wang F., Li G., Li Y., Xing L. (2020). Plasma IP-10 and MCP-3 levels are highly associated with disease severity and predict the progression of COVID-19. J. Allergy Clin. Immunol..

[33] Ackermann M., Verleden S.E., Kuehnel M., Haverich A., Welte T., Laenger F., Vanstapel A., Werlein C., Stark H., Tzankov A. (2020). Pulmonary Vascular Endothelialitis, Thrombosis, and Angiogenesis in Covid-19. N. Engl. J. Med..

[34] Kitajima D., Kasamatsu A., Nakashima D., Miyamoto I., Kimura Y., Saito T., Suzuki T., Endo-Sakamoto Y., Shiiba M., Tanzawa H. (2016). Tie2 Regulates Tumor Metastasis of Oral Squamous Cell Carcinomas. J. Cancer.

[35] Craver B.M., El Alaoui K., Scherber R.M., Fleischman A.G. (2018). The Critical Role of Inflammation in the Pathogenesis and Progression of Myeloid Malignancies. Cancers.

[36] Cervantes-Villagrana R.D., Albores-Garcia D., Cervantes-Villagrana A.R., Garcia-Acevez S.J. (2020). Tumor-induced neurogenesis and immune evasion as targets of innovative anti-cancer therapies. Signal Transduct. Target. Ther..

[37] Chen M., Wu Y., Jia W., Yin M., Hu Z., Wang R., Li W., Wang G. (2020). The predictive value of serum amyloid A and C-reactive protein levels for the severity of coronavirus disease 2019. Am. J. Transl. Res..

[38] Sack G.H. (2018). Serum amyloid A–A review. Mol. Med..

[39] Abbas A.K. (2020). The Surprising Story of IL-2: From Experimental Models to Clinical Application. Am. J. Pathol..

[40] Hu J.Y., Li G.C., Wang W.M., Zhu J.G., Li Y.F., Zhou G.H., Sun Q.B. (2002). Transfection of colorectal cancer cells with chemokine MCP-3 (monocyte chemotactic protein-3) gene retards tumor growth and inhibits tumor metastasis. World J. Gastroenterol..

[41] Okada M., Saio M., Kito Y., Ohe N., Yano H., Yoshimura S., Iwama T., Takami T. (2009). Tumor-associated macrophage/microglia infiltration in human gliomas is correlated with MCP-3, but not MCP-1. Int. J. Oncol..

[42] Corrales L., McWhirter S.M., Dubensky T.W., Gajewski T.F. (2016). The host STING pathway at the interface of cancer and immunity. J. Clin. Investig..

[43] Snell L.M., McGaha T.L., Brooks D.G. (2017). Type I Interferon in Chronic Virus Infection and Cancer. Trends Immunol..

[44] Corren J., Ziegler S.F. (2019). TSLP: From allergy to cancer. Nat. Immunol..

[45] Tokunaga R., Zhang W., Naseem M., Puccini A., Berger M.D., Soni S., McSkane M., Baba H., Lenz H.J. (2018). CXCL9, CXCL10, CXCL11/CXCR3 axis for immune activation–A target for novel cancer therapy. Cancer Treat. Rev..

[46] Niyongere S., Lucas N., Zhou J.M., Sansil S., Pomicter A.D., Balasis M.E., Robinson J., Kroeger J., Zhang Q., Zhao Y.L. (2019). Heterogeneous expression of cytokines accounts for clinical diversity and refines prognostication in CMML. Leukemia.

[47] Garner H., de Visser K.E. (2020). Immune crosstalk in cancer progression and metastatic spread: A complex conversation. Nat. Rev. Immunol..

[48] Saini G., Aneja R. (2021). Cancer as a prospective sequela of long COVID-19. BioEssays News Rev. Mol. Cell. Dev. Biol..

[49] Li G., Fan Y., Lai Y., Han T., Li Z., Zhou P., Pan P., Wang W., Hu D., Liu X. (2020). Coronavirus infections and immune responses. J. Med. Virol..

[50] Crook H., Raza S., Nowell J., Young M., Edison P. (2021). Long covid—mechanisms, risk factors, and management. BMJ.

[51] Herzog S., De Bie J., Abrams S., Wouters I., Ekinci E., Patteet L., Coppens A., De Spiegeleer S., Beutels P., Van Damme P. (2021). Seroprevalence of IgG antibodies against SARS coronavirus 2 in Belgium–A serial prospective cross-sectional nationwide study of residual samples. medRxiv.

