# Supplementary material for: Blood Cytokine Analysis Suggests That SARS-CoV-2 Infection Results in a Sustained Tumour Promoting Environment in Cancer Patients
Source: Cancers (Basel). 2021 Nov 15;13(22):5718. doi: 10.3390/cancers13225718 (PMC8616215; doi:10.3390/cancers13225718)
Supplement: Supplementary file 1 [file cancers-13-05718-s001.zip › cancers-1456604-supplementary.pdf]

Supplementary Table S1: Patient characteristics*

	Solid cancer (n = 68)		Haematological cancer (n = 38)		HCW (n = 57)	
	n (%) or mean (SD)		n (%) or mean (SD)		n (%) or mean (SD)	
	**Exposed[a]**	**Unexp[b]**	**Exposed[a]**	**Unexp[b]**	**Exposed[a]**	**Unexp[b]**
Sleep apnoea	3 (8.3%)	1 (3.1%)	1 (6.3%)	0 (0%)	0 (0%)	1 (2.4%)
Cardiovascular disease	3 (8.3%)	6 (18.8%)	2 (12.5%)	3 (9.4%)	1 (6.7%)	0 (0%)
Thromboembolic disease	2 (5.6%)	3 (9.4%)	2 (12.5%)	1 (3.1%)	0 (0%)	0 (0%)
Renal disease	1 (2.8%)	1 (3.1%)	1 (6.3%)	0 (0%)	0 (0%)	0 (0%)
Pulmonary disease	7 (19.4%)	2 (6.3%)	4 (25.0%)	1 (3.1%)	4 (26.7%)	3 (7.1%)
Diabetes	6 (16.7%)	2 (6.3%)	1 (6.3%)	0 (0%)	0 (0%)	0 (0%)
Metabolic disease	4 (11.1%)	3 (9.4%)	1 (6.3%)	2 (6.3%)	1 (6.7%)	3 (7.1%)
Hypertension	6 (16.7%)	5 (15.6%)	3 (18.8%)	3 (9.4%)	1 (6.7%)	1 (2.4%)
Infection	5 (13.9%)	6 (18.8%)	4 (25.0%)	7 (21.9%)	0 (0%)	2 (4.8%)
Allergic constitution	2 (5.6%)	0 (0%)	0 (0%)	1 (3.1%)	1 (6.7%)	3 (7.1%)
Gastrointestinal disease	2 (5.6%)	6 (18.8%)	4 (25.0%)	3 (9.4%)	0 (0%)	4 (9.5%)
Autoimmune disorder	3 (8.3%)	0 (0%)	1 (6.3%)	1 (3.1%)	1 (6.7%)	2 (4.8%)
Recent non-COVID-19 vaccination	0 (0%)	1 (3.1%)	0 (0%)	0 (0%)	0 (0%)	0 (0%)
Epileptic & neurologic disorder	1 (2.8%)	1 (3.1%)	1 (6.3%)	1 (3.1%)	1 (6.7%)	0 (0%)
Bone disorders	0 (0%)	0 (0%)	0 (0%)	1 (3.1%)	0 (0%)	0 (0%)

* Consult Table 1 in main text for other patient variables
HCWs: Health care workers
SD: standard deviation
[a]All exposed individuals were selected
[b]Unexposed individuals were group-matched to exposed individuals based on age, gender and cancer type in case of cancer patients

Supplementary Table S2: Overview of all patients with information on cancer types, topography, morphology and behaviour of the malignancy. Last column represents the exposure to SARS-CoV-2 confirmed by either PCR or positive serology.

	Cancer type	Topography	Morphology	Behaviour	Gender	Age	SARS-CoV-2-Exposed
**Carcinomas**							
**Head and neck cancer**	Squamous cells head and neck cancer	C01.9	8070	3	M	57	Yes
	Malignant neoplasm of ventral surface of tongue	C02.2	NA	NA	M	72	Yes
	Squamous cells in tonsils	C09.8	8070	3	M	56	Yes
	Squamous cells in oropharynx	C10.9	8070	3	F	69	No
	Squamous cells in oropharynx	C10.9	8070	3	M	64	Yes
	Squamous cells in oropharynx, HPV-positive	C10.9	8085	3	M	67	Yes
	Squamous cells in nasopharynx	C11.9	8070	3	M	54	No
	Epithelial carcinoma in nasopharynx	C11.9	8010	3	M	67	Yes
	Squamous cells in hypopharynx	C13.9	8070	3	M	66	No
**Gastric cancer**	Malignant neoplasm of esophagus	C15.1	NA	NA	F	69	Yes
	Squamous cell carcinoma in esophagus, also malignancy in lower respiratory tract and prostate	C15.5	8070	3	M	62	Yes
	Stromal carcinoma in the pyloric antrum	C16.3	8936	3	F	75	Yes
	Adenocarcinoma of the gastric cardium	C16.0	8144	3	F	72	Yes
	Malignant neoplasm of ceacum	C18.0	NA	3	F	71	Yes
	Combined carcinoid and adenocarcinoid of ascending colon	C18.2	8244	3	M	68	Yes
	Adenocarcinoma of ascending colon	C18.2	8140	3	F	76	Yes
	Adenocarcinoma of sigmoid colon	C18.7	8140	3	M	49	No
	Adenocarcinoma of rectosigmoid junction	C19.9	8140	3	M	63	Yes
	Adenocarcinoma of rectum	C20.9	8140	3	M	63	Yes
**Hepatobiliary cancer**	Intrahepatic bile duct cholangiocarcinoma	C22.1	8160	3	F	65	No
	Extrahepatic bile duct cholangiocarcinoma	C24.0	8160	3	F	54	Yes
**Pancreatic cancer**	Adenocarcinoma of head of pancreas	C25.0	8140	3	M	56	No
	Adenocarcinoma of body of pancreas	C25.1	8140	3	M	67	Yes
**Respiratory tract cancer**	Squamous cell carcinoma of the glottis, also prostate cancer	C32.0	8070	3	M	72	No
	Squamous cell and atypical carcinoid tumour of the lung	C34.1	8249	3	M	68	No
	Squamous cell carcinoma of the lung	C34.1	8070	3	M	67	No
	Adenocarcinoma of the lung	C34.9	8140	3	M	67	Yes
	Thymoma NOS	C37.9	8580	3	F	56	Yes
	Malignant neoplasm of thymus	C37.9	NA	NA	F	60	Yes
**Breast cancer**	Secretory carcinoma of central portion of breast	C50.1	8502	3	F	75	Yes
	Intraductal and lobular carcinoma of central portion of breast	C50.1	8522	3	F	54	Yes
	Intraductal carcinoma of upper-inner quadrant of breast	C50.2	8500	3	F	54	No
	Intraductal carcinoma of upper-outer quadrant of breast	C50.4	8500	3	F	61	Yes
	Intraductal carcinoma of upper-outer quadrant of breast	C50.4	8500	3	F	60	No
	Intraductal carcinoma of upper-outer quadrant of breast	C50.4	8500	3	F	35	Yes
	Intraductal carcinoma of upper-outer quadrant of breast	C50.4	8500	3	F	62	Yes
	Intraductal carcinoma of upper-outer quadrant of breast	C50.4	8500	3	F	63	No
	Intraductal carcinoma of upper-outer quadrant of breast	C50.4	8500	3	F	44	No
	Lobular carcinoma of upper-outer quadrant of breast	C50.4	8520	3	F	55	Yes
	Intraductal carcinoma of overlapping sites of breast	C50.8	8500	3	F	65	No
	Intraductal carcinoma of breast of unspecified site	C50.9	8500	3	F	52	No
	Intraductal carcinoma of breast of unspecified site	C50.9	8500	3	F	66	No
	Intraductal carcinoma of breast of unspecified site	C50.9	8500	3	F	57	Yes
	Intraductal carcinoma of breast of unspecified site	C50.9	8500	3	F	42	Yes
	Intraductal carcinoma of breast of unspecified site	C50.9	8500	3	F	42	No

**Female reproductive organ cancer**	Adenocarcinoma of the endocervix	C53.0	8140	3	F	47	Yes
	Squamous cells of the exocervix	C53.1	8070	3	F	43	No
	Adenocarcinoma of the endometrium	C54.1	8380	3	F	73	Yes
	Ovarian cancer	C56	NA	NA	F	60	No
**Prostate cancer**	Malignant neoplasm of prostate	C61	NA	NA	M	70	No
	Adenocarcinoma of the prostate	C61.9	8140	3	M	66	No
	Adenocarcinoma of the prostate	C61.9	8140	3	M	60	No
	Adenocarcinoma of the prostate	C61.9	8140	3	M	84	No
	Malignant neoplasm of prostate	C61.9	NA	NA	M	80	Yes
**Kidney cancer**	clear cell mesonephroid carcinoma of the kidney	C64.9	8310	3	F	70	No
	clear cell mesonephroid carcinoma of the kidney	C64.9	8310	3	M	53	Yes
	clear cell mesonephroid carcinoma of the kidney	C64.9	8310	3	M	53	No
**Bladder cancer**	large cell neuroendocrine carcinoma of the bladder	C67.9	8013	3	F	67	Yes
**Brain cancer**	Glioblastoma in the temporal lobe	C71.2	9440	3	M	52	No
	Carcinoma in the pineal gland	C75.3	NA	NA	M	16	Yes
**Sarcomas and melanomas**							
**Mesothelioma**	Epitheloid mesothelioma	C38.4	9052	3	F	64	No
	Biphasic mesothelioma	C38.4	9053	3	M	76	No
	Unclassified Mesothelioma	C45.0	NA	NA	M	69	No
**Other sarcomas**	Tenosynoval giant cell tumour in the short bones of the lower limb	C40.3	9252	NA	M	32	Yes
	Differentiated liposarcoma of the peritoneum	C48.0	8858	3	F	51	Yes
	Squamous cell carcinoma of other connective and soft tissue	C49	8070	3	M	73	Yes
	Ewing's sarcoma	C49.2	9260	3	M	17	No
	Reticulum cell sarcoma	C69.4	9591	3	F	70	No
**Melanoma**	Malignant melanoma	C44.5	8720	3	F	58	No
	Superficial spreading melanoma	C44.4	8743	3	M	88	No
	Malignant melanoma of the eye	C69.4	8720	3	F	36	Yes
**Haematological malignancies**							
**Lymphoma**	Mantle cell lymphoma	C07.9	9673	3	F	66	Yes
	Burkitt Lymphoma	C34.1	9680	3	F	32	No
	Thymic large B-cell lymphoma	C37.9	9679	3	M	59	No
	Mantle cell lymphoma	C38.1	9673	3	F	52	Yes
	B-cell lymphoma in the splenic marginal zone	C42.1	9689	3	M	82	No
	B-cell lymphoma in the splenic marginal zone	C42.2	9689	3	F	47	Yes
	B-lymphoblastic lymphoma	C42.1	9811	3	M	30	No
	B-lymphoblastic lymphoma	C42.1	9811	3	M	6	No
	B-lymphoblastic lymphoma with hyper diploidy	C42.1	9815	3	F	26	Yes
	B-lymphoblastic lymphoma with hyper diploidy	C42.1	9815	3	F	26	No
	Hodgkin lymphoma	C50	NA	NA	F	54	Yes
	Hodgkin lymphoma	C77.1	9659	3	F	56	Yes
	Burkitt lymphoma	C77.8	9680	3	M	45	No
	Follicular lymphoma	C77.8	9690	3	F	68	No
	Burkitt lymphoma	C77.9	9680	3	M	71	No
	Diffuse large B-cell lymphoma	C83.3	9680	3	M	37	yes
	Diffuse large B-cell lymphoma	C83.3	9680	3	V	62	yes
**Multiple Myeloma**	Multiple myeloma	C42	9732	3	F	75	Yes
	Multiple myeloma	C42.1	9732	3	M	58	Yes
	Multiple myeloma	C42.1	9732	3	F	53	No
	Multiple myeloma	C 90.0	NA	NA	M	63	yes
	Multiple myeloma	C 90.0	NA	NA	M	58	yes

**Leukaemia**	B-cell chronic lymphocytic leukemia	C42.1	9823	3	F	65	No
	Precursor T-cell lymphoblastic leukemia	C42.1	9837	3	M	37	No
	Acute myeloid leukemia	C42.1	9861	3	M	54	No
	Chronic myeloid leukemia, BCR/ABL-positive	C42.1	9875	3	F	23	No
	Chronic myeloid leukemia, BCR/ABL-positive	C92.1	9875	3	M	69	Yes
	Chronic myelomonocytic leukemia	C42.1	9945	3	M	68	No
	Primary myelofibrosis	C42.1	9961	3	M	51	Yes
	Myoproliferative neoplasm unclassifiable	C42.1	9975	3	F	66	No
**Non-cancerous**	Refractory anemia with ring sideroblasts	C42.1	9982	3	F	72	Yes
	Refractory anemia with excess blasts	C42.1	9983	3	M	54	No
	Refractory anemia with excess blasts	C42.1	9983	3	M	68	No
	Refractory anemia with excess blasts	C42.1	9983	3	F	65	No
	Refractory cytopenia with multilineage displasia	C42.1	9985	3	M	52	No

Supplementary Table S3: Alterations in CCGs for unexposed solid and haematological malignancy patients compared to unexposed healthcare workers (HCWs). The table represents the p-values and fold changes with one-way ANOVA between the three groups followed by pair wise comparisons with post-hoc Tukey correction for multiple comparisons.

		Solid cancer vs HCW		Haematological cancer vs HCW	
CCG upregulated	*P* ANOVA	Fold Change Increase (95% CI)	*P* Tukey	Fold Change Increase (95% CI)	*P* Tukey
TNF-α	< 0.001**	1.3 (1.2 - 1.5)	< 0.001	1.3 (1.1-1.5)	0.002
IL-6	< 0.001**	1.9 (1.6 - 2.3)	< 0.001	1.6 (1.3-2.0)	< 0.001
IL-1Ra	0.002**	1.6 (1.2 - 2.1)	0.003	1.5 (1.1-2.0)	0.04
IL-8	0.008**	1.8 (1.3 - 2.7)	0.007	1.5 (0.9-2.2)	ns
CRP	0.002**	2.3 (1.5 - 3.6)	0.002	1.3 (0.8-2.2)	ns
SAA	< 0.001**	2.6 (1.6 - 4.1)	< 0.001	1.2 (0.7-2.0)	ns
IFN-γ	< 0.001**	5.1 (3.3 - 7.9)	< 0.001	8.1 (5.0-13.2)	< 0.001
IL-18	0.029**	1.3 (1.0-1.7)	ns	1.5 (1.1-2.0)	0.04
IL-17A	< 0.001**	1.8 (1.5 - 2.2)	< 0.001	1.7 (1.3-2.1)	< 0.001
IL-22	< 0.001**	1.2 (1.1 - 1.4)	0.011	1.3 (1.1-1.5)	0.001
IL-33	0.002**	1.1 (1.0 - 1.2)	0.003	1.1 (1.0-1.1)	0.032
IL-5	0.006**	1.4 (1.1 - 1.6)	0.005	1.1 (0.9-1.4)	ns
TSLP	< 0.001**	1.8 (1.5 - 2.1)	< 0.001	1.4 (1.1-1.7)	0.011
IL-9	< 0.001**	1.4 (1.2 - 1.6)	< 0.001	1.2 (1.1-1.4)	0.013
IL-21	0.017**	1.4 (1.1 - 1.9)	0.018	1.3 (1.0-1.7)	ns
Eotaxin	0.043	1.3 (1.1 - 1.7)	0.037	1.1 (0.9-1.4)	ns
IL-10	< 0.001**	1.3 (1.2 - 1.4)	< 0.001	1.2 (1.1-1.4)	< 0.001
IL-2Rα	0.002**	1.5 (1.2-1.9)	0.002	1.3 (1.0-1.7)	ns
IL-15	0.004**	1.2 (1.0-1.4)	0.032	1.3 (1.1-1.5)	0.009
VEGF-A	0.039	1.8 (1.2-3.0)	0.034	1.3 (0.7-2.1)	ns
bFGF	0.056	2.7 (1.2-6.0)	0.048	1.4 (0.6-3.5)	ns
PlGF	< 0.001**	1.4 (1.2-1.7)	0.001	1.5 (1.2-1.8)	0.002
Flt-1	0.027**	1.3 (1.1-1.6)	0.031	1.2 (1.0-1.5)	ns
EPO	< 0.001**	2.1 (1.5-2.8)	< 0.001	2.3 (1.6-3.2)	< 0.001
sICAM-1	0.017**	1.1 (1.0-1.1)	0.031	1.1 (1.0-1.1)	ns
G-CSF	< 0.001**	1.8 (1.4-2.4)	< 0.001	1.7 (1.2-2.2)	0.003
M-CSF	< 0.001**	1.5 (1.2-1.8)	0.001	1.3 (1.1-1.7)	0.033
GM-CSF	0.015**	1.1 (1.0-1.1)	0.015	1.0 (1.0-1.1)	ns
MCP-1	< 0.001**	1.7 (1.4-2.0)	< 0.001	1.6 (1.4-1.9)	< 0.001

**MCP-2**	0.003**	1.3 (1.1-1.6)	0.024	1.4 (1.1-1.8)	0.007
**MCP-3**	0.012**	1.3 (1.1-1.6)	0.01	1.1 (0.9-1.3)	ns
**MIP-1β**	0.035	1.4 (1.1-1.9)	0.036	1.3 (0.9-1.7)	ns
**MIP-3α**	0.013**	1.5 (1.1-1.9)	0.012	1.1 (0.8-1.5)	ns
**Fractalkine**	< 0.001**	1.3 (1.1-1.5)	0.004	1.3 (1.1-1.5)	0.003
**CTACK**	0.004**	1.29 (1.1-1.5)	0.009	1.27 (1.1-1.5)	0.033

**CCG downregulated**	***P* ANOVA**	**Fold Change Decrease (95% CI)**	***P* Tukey**	**Fold Change Decrease (95% CI)**	***P* Tukey**
**Tie-2**	<0.001**	1.4 (1.2-1.5)	<0.001	1.3 (1.1-1.5)	0.001

HCW: Health care workers

**FDR-corrected significance (q-value) <0.05

Supplementary Table S4: Alterations in CCGs levels in SARS-CoV-2-exposed individuals compared to unexposed individuals. A) healthcareworkers (HCWs), B) solid cancer patients and C) haematological malignancy patients. T-test was performed on Log10 transformed data.

A. Health care workers (exposed versus non-exposed)

Upregulated CCG	Fold increase (95% CI)	*P* value
**TNF-α**	1.4 (1.2-1.7)	< 0.001**
**IL-6**	1.2 (1.0-1.3)	0.011
**IL-1Ra**	2.0 (1.4-2.9)	0.001**
**SAA**	1.5 (1.0-2.0)	0.032
**IFN-γ**	11.8 (5.6-25.0)	< 0.001**
**IL-18**	1.4 (1.0-2.0)	0.0496
**IL-17F**	3.0 (1.1-8.1)	0.039
**IL-33**	1.2 (1.1-1.3)	< 0.001**
**IL-13**	1.6 (1.1-2.3)	0.009**
**IL-5**	1.2 (1.0-1.3)	0.046
**TSLP**	1.2 (1.0-1.5)	0.044
**IL-21**	1.8 (1.2-2.7)	0.005**
**IL-10**	1.1 (1.0-1.2)	0.003**
**IL-2**	1.9 (1.1-3.4)	0.032
**G-CSF**	1.4 (1.1-1.8)	0.004**
**GM-CSF**	1.1 (1.0-1.2)	0.031
**MCP-1**	1.4 (1.1-1.7)	0.003**

Downregulated CCG	Fold decrease (95% CI)	*P* value
**IP-10**	1.5 (1.2-2.0)	0.004**
**PlGF**	1.3 (1.0-1.7)	0.048
**Tie-2**	1.7 (1.4-2.1)	< 0.001**

B. Solid cancer patients (exposed versus non-exposed)

Upregulated CCG	Fold increase (95% CI)	*P* value
**SAA**	2.5 (1.1-5.6)	0.026
**CRP**	2.1 (1.1-4.0)	0.035
**IL-2**	1.9 (1.2-3.0)	0.006
**MCP-3**	1.3 (1.1-1.7)	0.008

Downregulated CCG	Fold decrease (95% CI)	*P* value
**BDNF**	3.7 (1.7-8.0)	0.002
**bFGF**	2.9 (1.3-6.3)	0.012
**VEGF-C**	1.9 (1.2-3.0)	0.011
**Total TGF-β**	1.8 (1.0-3.1)	0.047
**IL-9**	1.2 (1.0-1.4)	0.028

C. Haematological patients (exposed versus non-exposed)

Upregulated CCG	Fold increase (95% CI)	*P* value
**SAA**	3.1 (1.4-6.9)	0.010**
**IFN-β**	2.7 (1.3-5.6)	0.011
**IP-10**	2.5 (1.5-4.4)	0.002**
**CRP**	2.4 (1.2-4.7)	0.02
**TSLP**	1.4 (1.1-1.9)	0.017**
**TNF-α**	1.3 (1.0-1.5)	0.032**
**sVCAM-1**	1.1 (1.0-1.2)	0.017**

* T-test was performed on log10 transformed data.

** FDR-corrected significance (q-value) <0.05

	Solid cancer (n= 45) (n(%))	Haematological cancer (n = 17) (n(%))	HCWs (n = 19) (n(%))	statistics*
**Asymptomatic**	10 (22.2%)	5 (29.4%)	7 (36.8%)	ns
**Mild**	19 (42.2%)	7 (41.2%)	10 (52.6%)	ns
**Moderate**	3 (6.7%)	2 (11.8%)	0 (0%)	ns
**Severe**	8 (17.8%)	2 (11.8%)	2 (10.5%)	ns
**Critical**	1 (2.2%)	1 (5.9%)	0 (0%)	ns
**Death**	4 (8.9%)	0 (0%)	0 (0%)	ns
* Fisher's exact test				

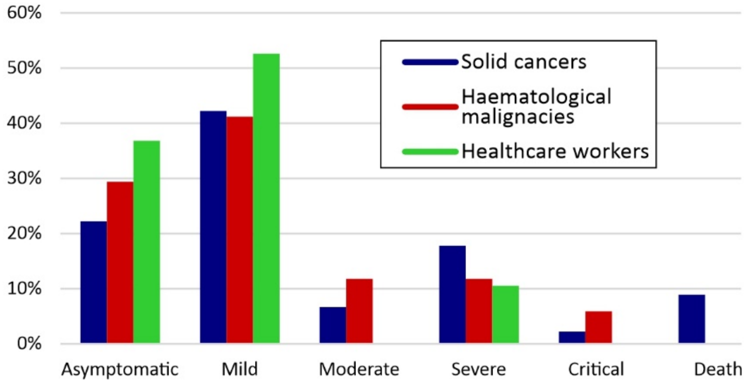

Supplementary Figure S1: Proportion of patients according to severity in solid and haematological malignancies as well as HCWs. The upper panel shows the precise numbers and the statistics.

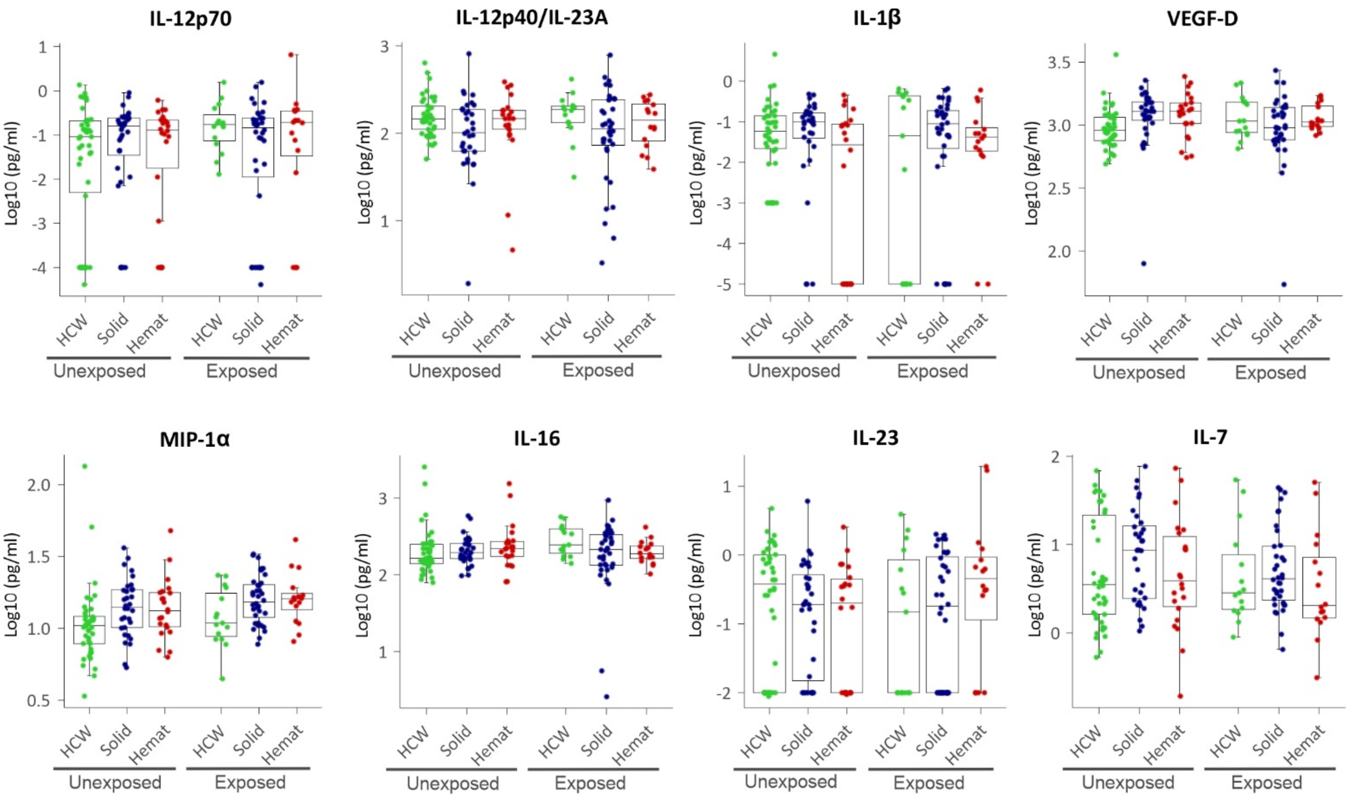

Supplementary Figure S2. Plasma levels of additional CCGs. HCW, health care workers (green), Solid, patients with solid tumours (blue), Hemat, patients with haematological malignancies (red). Each dot represents the sample closest to exposure in case of exposed patients, and an average of multiple timepoints, when available, for unexposed patients.

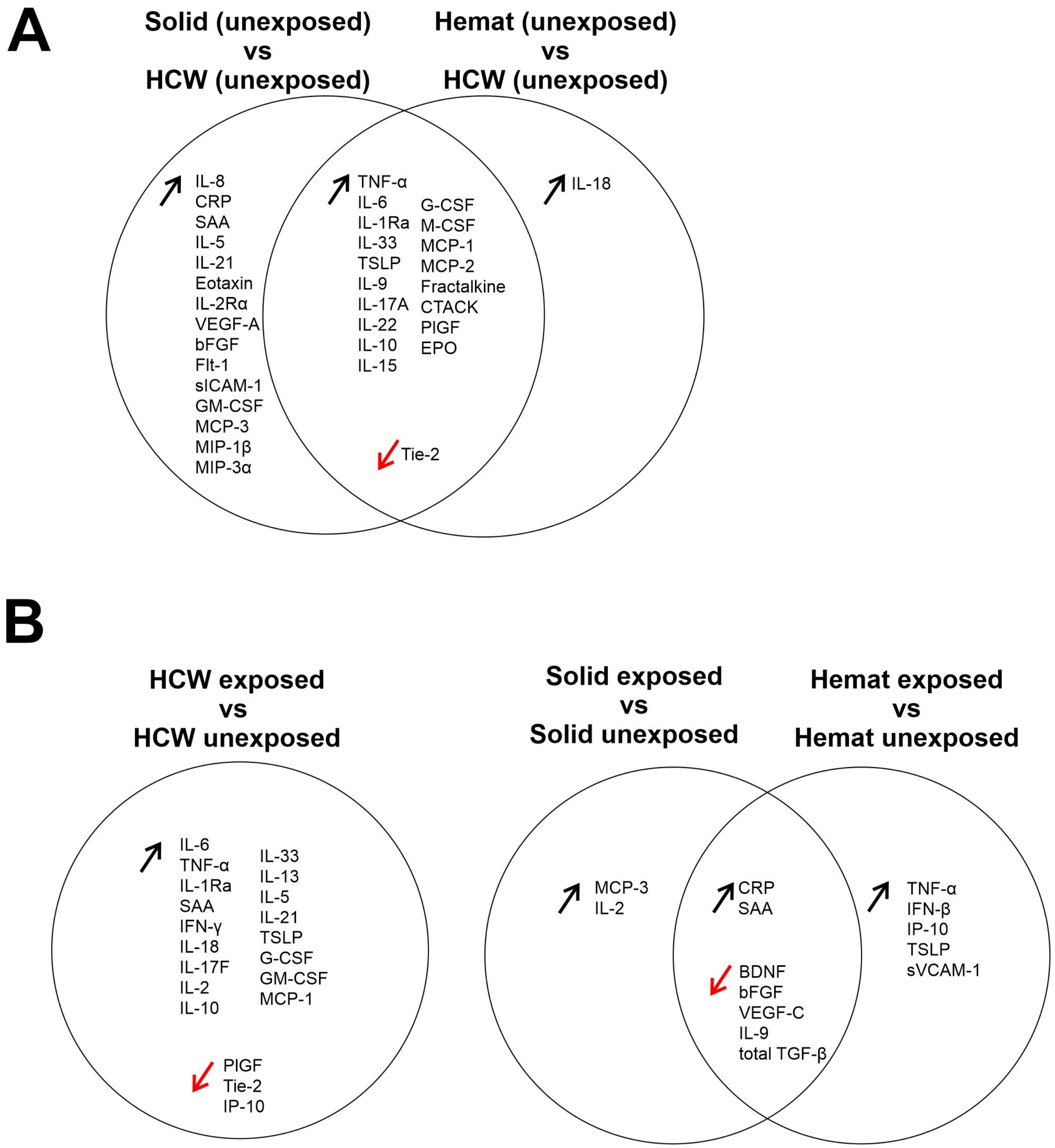

Supplementary Figure S3: Summarising figure showing cytokines significantly increased and decreased **A)** in relatively unexposed patients with solid cancers and with haematological malignancies compared to unexposed HCWs. **B)** Cytokines significantly increased and decreased in HCWs exposed to SARS-CoV-2 compared to unexposed HCWs and increased and decreased in SARS-CoV-2 exposed solid and haematological malignancy patients compared to

unexposed solid and haematological malignancy patients. HCW, health care workers, Solid, patients with solid tumours, Hemat, patients with haematological malignancies.
